# Sex-differences in COVID-19 associated excess mortality is not exceptional for the COVID-19 pandemic

**DOI:** 10.1038/s41598-021-00213-w

**Published:** 2021-10-21

**Authors:** Jens Nielsen, Sarah K. Nørgaard, Giampaolo Lanzieri, Lasse S. Vestergaard, Kaare Moelbak

**Affiliations:** 1 Infectious Disease Epidemiology and Prevention, Statens Serum Institut, Copenhagen, Denmark; 2 Statistical Office of the European Union (Eurostat), Population and Migration Unit, Luxembourg, Luxembourg; 3 Department of Veterinary and Animal Sciences, Faculty of Health and Medical Sciences, University of Copenhagen, Copenhagen, Denmark

**Keywords:** Risk factors, Epidemiology

## Abstract

Europe experienced excess mortality from February through June, 2020 due to the COVID-19 pandemic, with more COVID-19-associated deaths in males compared to females. However, a difference in excess mortality among females compared to among males may be a more general phenomenon, and should be investigated in none-COVID-19 situations as well. Based on death counts from Eurostat, separate excess mortalities were estimated for each of the sexes using the EuroMOMO model. Sex-differential excess mortality were expressed as differences in excess mortality incidence rates between the sexes. A general relation between sex-differential and overall excess mortality both during the COVID-19 pandemic and in preceding seasons were investigated. Data from 27 European countries were included, covering the seasons 2016/17 to 2019/20. In periods with increased excess mortality, excess was consistently highest among males. From February through May 2020 male excess mortality was 52.7 (95% PI: 56.29; 49.05) deaths per 100,000 person years higher than for females. Increased male excess mortality compared to female was also observed in the seasons 2016/17 to 2018/19. We found a linear relation between sex-differences in excess mortality and overall excess mortality, i.e., 40 additional deaths among males per 100 excess deaths per 100,000 population. This corresponds to an overall female/male mortality incidence ratio of 0.7. In situations with overall excess mortality, excess mortality increases more for males than females. We suggest that the sex-differences observed during the COVID-19 pandemic reflects a general sex-disparity in excess mortality.

## Introduction

Through spring 2020, Europe experienced high excess mortality caused by the spread of the severe acute respiratory syndrome coronavirus 2 (SARS-CoV-2) and the associated coronavirus disease (COVID-19). As documented by the network for European monitoring of excess mortality for public health action (EuroMOMO) the pooled European mortality started increasing from 2020-W08 (ISOweek 8601 definition and format^1^) and peaked in 2020-W14^2^, with a pooled all-cause excess number of 35,802 deaths in the 26 participating countries^3^.

Worldwide, a higher number of deaths among confirmed COVID-19 cases have been reported for males than for females resulting in a sex-difference in COVID-19 case fatalities^4–6^ and in all-cause mortality^7^, which may be understood as a particular feature of the current pandemic. It is, however, widely recognized that there is a general underlying difference between females and males in mortality and morbidity e.g. resulting in a longer life expectancy for females^8^. This is particularly seen in Western countries, where males generally die earlier than females^9^. Interactions between sex hormones and the immune system, causing different sex patterns in immune responses may play a role^10–12^, although physiological factors and gender-related behavior may also be of importance^13–15^.

Furthermore, sex-differences in excess mortality have also been reported for other infectious diseases causing elevated excess mortality^16,17^ also in situations with extreme mortality^18^. While sex-differences in general mortality has been investigated, few studies have investigated sex-differential excess all-cause mortality, by which we mean differences in excess mortality among females compared to excess mortality among males.

When investigating sex-differences in mortality due to COVID-19, or any other infection, it is important to recognize that reported deaths associated to the infection may be biased e.g. due to non-random testing, misclassification or lack of consistent reporting of death certificates. To overcome this bias, we applied population based analyses of sex-differential excess mortality i.e. excess relative to expected baseline mortalities for each sex.

## Data and methods

We estimated sex specific all-cause excess mortality in the seasons 2016/17 to 2019/20 (week 27 to week 26 the following year), to investigate sex-differential excess mortality during the first half year of the COVID-19 pandemic and in previous periods with excess mortality. For this purpose, we applied the EuroMOMO model, a time series Poisson regression accounting for trend and seasonality^19^, on data from each of the European countries reporting all-cause death counts to the statistical office of the European Union, Eurostat. Expected all-cause mortality and excess all-cause mortality were estimated separately for each sex. Estimates of excess mortality from the countries was pooled^19^ i.e. excess mortalities are stratified by country, thus accounting for disparities between the countries.

### Data sources

Data on all-cause death counts, from a number of European countries, are freely downloadable from Eurostat^20^, by NUTS-code^21^, sex, 5-years age groups and ISOweek.

Population size on January 1st for each of the countries are also freely available from Eurostat^20^, by NUTS-code, sex and age.

Data used in this study were downloaded November 4, 2020.

### Data preparation and criteria for inclusion

#### Criteria for inclusion

In order to apply the EuroMOMO model to estimate the expected baseline number of deaths in one winter season (week 27 to week 26 the following year), historical data for the preceding five seasons are required. Therefore, for each season we limited the analyses to countries having data covering at least the previous five seasons before and the full actual season. Further, all analyses were performed on country level (NUTS level 0), and aggregated to the following age groups: 0–14, 15–44, 45–64, 65–74, 75–84, 85 + years and all ages. Analyses were limited to countries with data available on age and sex.

#### EuroMOMO input data

The EuroMOMO model requires input data to be one record for each death, even though it is aggregated by ISOweek. Therefore, downloaded weekly death counts from Eurostat were randomly distributed over the week to fit the EuroMOMO model. This could be done without loss of information.

#### Weekly population data

Population size by ISOweek were linearly interpolated from the January 1st population data, by country (NUTS level 0), sex and age group.

### Analyses

#### Expected and excess mortality

We used the EuroMOMO model R package^22^ to estimate the expected (baseline) weekly number of deaths for each country, by sex and age group, for each of the seasons 2015/16 to 2019/20. Number of countries with sufficient retrospective data dropped drastically for seasons before 2016/17 (Supplementary S1), why earlier seasons were not included in the analyses. The country baselines and excess number of deaths were pooled, stratified by country, to account for heterogeneity between counties, to provide European estimates of expected (baseline) number of deaths and excess number of deaths for each of the seasons^19^.

European weekly estimates of excess number of deaths i.e. difference between observed and expected numbers of deaths, as well as their variances were extracted from the pooled data. Furthermore, excess mortality incidences by 100,000 person years by sex and age group were calculated, using population data.

Differences in age-distribution between sexes may make the comparison between sexes on all ages misleading. Therefore, a weekly age standardized total over age groups according to the overall age group distribution of both sex was calculated.

#### Sex-differential excess mortality

Sex-differential excess mortality i.e. the differences in weekly female and male excess mortality incidences were expressed as the difference between female and male excess mortality incidence rates (female minus male). This measure eliminates the underlying difference in mortality between the sexes.

#### Sex-differential excess mortality versus overall excess mortality

A possible association between sex-differential excess mortality and overall excess mortality was explored by linear regression, using the respective excess mortalities for the seasons 2016/17 to and including the 2019/20 COVID-19 season.

## Results

Totally, 27 countries had sufficient data for estimation of baselines in two or more of the seasons 2016/17 to 2019/20 (Table 1). Of these, 25 countries had data for all 4 seasons, while France had data for the seasons 2018/19 and 2019/20, and Montenegro had data from 2016/17 to 2018/19. The included countries are listed in Supplementary S1.

**Table 1:** Number of deaths and populations, by season and gender.

Season*	Number of countries	Number of deaths (millions)	Population (millions)	Females		Males	
				Number of deaths (millions)	Population (millions)	Number of deaths (millions)	Population (millions)
2016/17	26	2.27	221.28	1.14	113.11	1.13	108.17
2017/18	26	2.27	221.75	1.13	113.33	1.14	108.42
2018/19	27	2.86	289.38	1.43	148.23	1.43	141.14
2019/20	26	2.92	289.44	1.45	148.24	1.47	141.20

*Week 27 to week 26 the following year.

**Figure 1:**
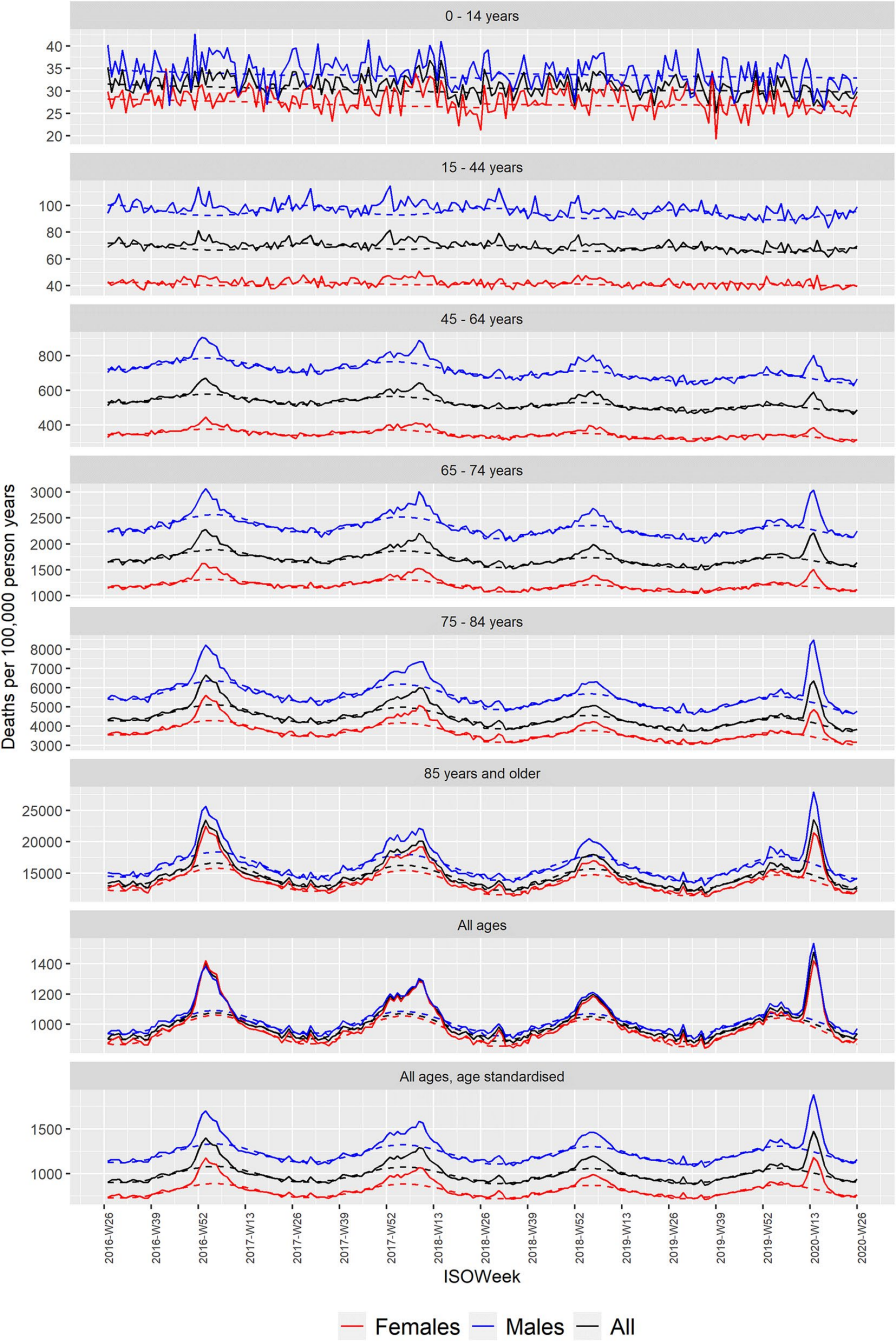
Pooled observed (solid) and expected (dashed) mortality rates by age group for the seasons 2015/16 to 2019/20.

Roughly, equal numbers of males and females died every season (Table 1), but females made up a larger proportion of the population. Hence, the estimated expected, underlying female mortality was lower than the expected underlying male mortality in all age groups (Fig. 1). There were virtually no differences in mortality between the sexes, when looking at crude all-age estimates; however, when investigating the sex-differences for all ages standardized according to the overall age group distribution of both sexes, the baseline mortalities for all ages reflect the underlying sex-differences in mortality observed in the age groups.

**Figure 2:**
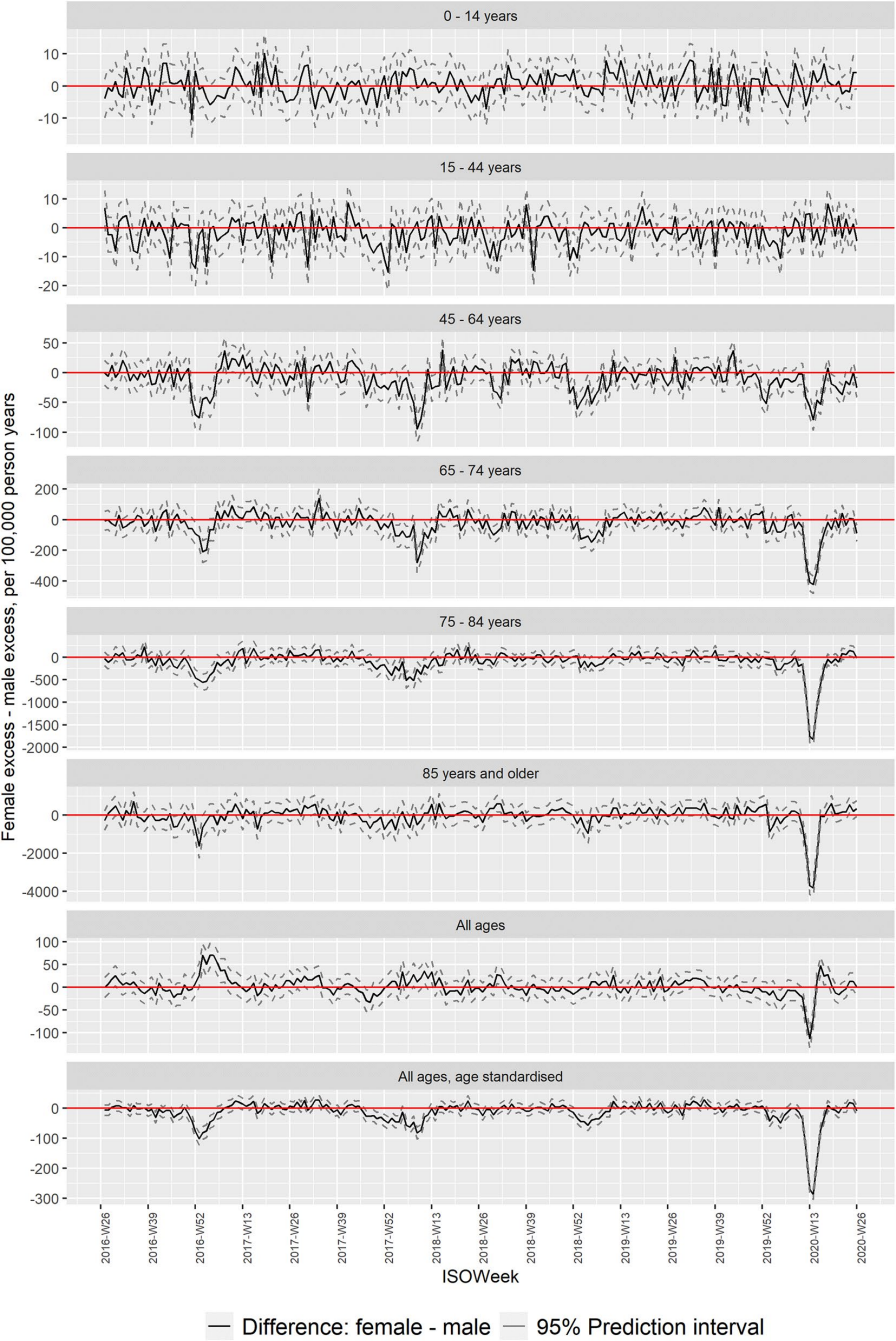
Pooled sex-differential excess mortality rates by age group for the seasons 2015/16 to 2019/20.

Comparing sex-differences in excess mortality, i.e. where the underlying baseline is excluded, the sex-differential, for each age group and for all ages combined (age-standardized), showed markedly lower excess mortalities for females during the COVID-19 pandemic (Fig. 2), most pronounced among individuals 45 years or older. Periods with markedly low female excess mortality compared to male were also observed in the seasons 2016/17 to 2018/19 (Fig. 2), while there were only sporadic periods of higher female excess mortality compared to male.

Periods with a significant sex-differential excess mortality, defined as periods with at least 3 coherent weeks with a weekly difference above 2 z-scores, occurred in all four seasons (Table 2), though with different duration and pattern (Fig. 2).

**Table 2:** Cumulated sex-differential excess mortality (females minus males).

Period	2016-W51 to 2017-W05	2017-W47 to 2018-W15	2018-W29 to 2019-W08	2019-W52 to 2020-W22
**Age group**	Female excess—male excess, deaths per 100,000 person years (95% Prediction Interval)			
0–14 years	− 3.09 (− 5.35; − 0.84)	0.42 (− 0.85; 1.70)	1.04 (0.15; 1.93)	0.38 (− 0.69; 1.44)
15–44 years	− 6.03 (− 8.36; − 3.70)	− 3.56 (− 4.91; − 2.21)	− 2.42 (− 3.36; − 1.47)	− 1.16 (− 2.30; − 0.02)
45–64 years	− 52.39 (− 61.10; − 43.67)	− 29.25 (− 34.00; − 24.49)	− 10.62 (− 13.72; − 7.51)	− 26.61 (− 30.27; − 22.96)
65–74 years	− 112.66 (− 139.72; − 85.59)	− 68.27 (− 83.28; − 53.26)	− 36.76 (− 46.70; − 26.81)	− 94.25 (− 106.28; − 82.21)
75–84 years	− 440.02 (− 507.22; − 372.81)	− 245.58 (− 282.27; − 208.89)	− 52.91 (− 76.27; − 29.55)	− 342.86 (− 370.14; − 315.59)
85 years and older	− 470.72 (− 705.46; − 235.98)	− 208.19 (− 332.31; − 84.06)	20.68 (− 62.47; 103.82)	− 494.90 (− 593.79; − 396.00)
All ages, age standardised	− 66.17 (− 73.99; − 58.36)	− 36.31 (− 40.60; − 32.02)	− 10.18 (− 13.16; − 7.20)	− 52.67 (− 56.29; − 49.05)

Periods with at least 3 coherent weeks with a weekly difference greater than 2 z-scores in at least one age group.

**Figure 3:**
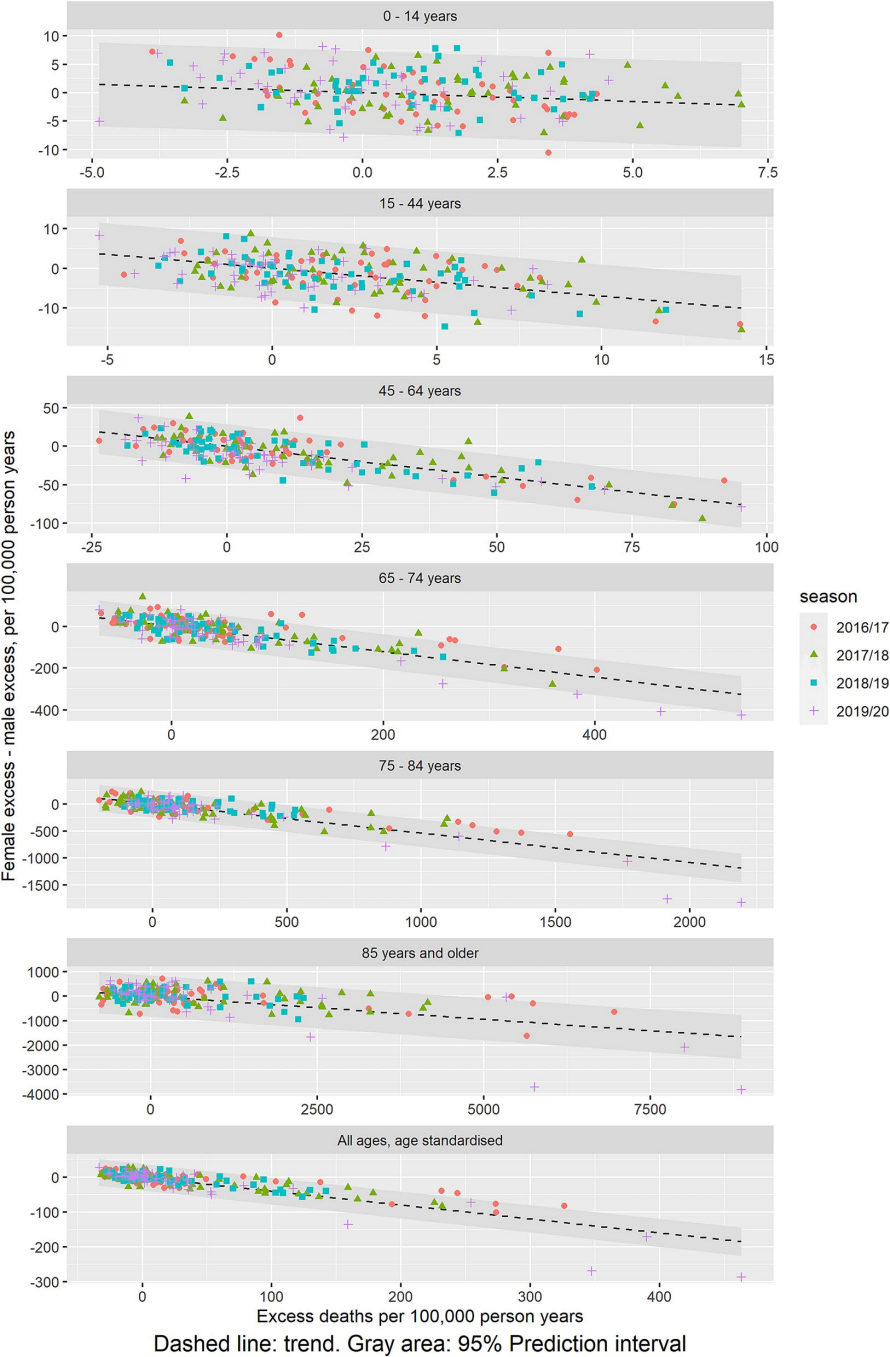
Relations between overall excess mortality and sex-differential excess mortality (female–male) for the seasons 2016/17 to 2019/20.

A potential relation between sex-differential excess mortality and overall excess mortality showed a significant linear trend towards an increasing higher difference between excess mortality for females and males with increasing overall excess mortality, with varying trends between age groups (Fig. 3). There are a one-to-one mapping between the linear trend (slope) and the female/male excess mortality incidence ratio (Supplementary S2). For example, the estimated overall age-standardised increase in the sex-difference of − 0.4 per increase in overall excess mortal. In other words, an increase in excess mortality of 100 per 100,000 population will be associated with 40 additional deaths among males. This corresponds to an overall age-standardised female/male mortality incidence ratio of 0.7 (Table 3). The female/male excess mortality incidence ratios suggests a u-shape with age, with highest disparity among 45–64 years old (ratio 0.4) and estimates approaching 1 at low and high ages (0.7 for children < 15 years and 0.8 for elderly >  = 85 years) (Table 3).

**Table 3:** Estimated linear association between sex-differential excess mortality and overall excess mortality, and corresponding female/male excess mortality incidence ratios.

Age group	Linear trends in females minus male excess mortality versus overall excess mortality (95% Confidence Interval)	Female/male excess mortality incidence ratio (95% Confidence Interval)
0 to 14 years	− 0.30 (− 0.52; − 0.08)	0.74 (0.59; 0.92)
15 to 44 years	− 0.70 (− 0.83; − 0.56)	0.48 (0.41; 0.56)
45 to 64 years	− 0.80 (− 0.88; − 0.72)	0.43 (0.39; 0.47)
65 to 74 years	− 0.61 (− 0.66; − 0.55)	0.54 (0.51; 0.57)
75 to 84 years	− 0.54 (− 0.58; − 0.50)	0.59 (0.57; 0.62)
≥ 85 years	− 0.19 (− 0.22; − 0.15)	0.83 (0.81; 0.86)
All ages, age standardized	− 0.4 (− 0.43; − 0.37)	0.67 (0.65; 0.69)

## Discussion

Our study support existing literature on all-cause sex-differential excess mortality in favor of females during periods with increased or extreme all-cause mortality. Further, we observed the sex-differential excess mortality to be of the same size, for all ages, 40 males per 100.000 population in absolute numbers, independent of what was causing the increased mortality; COVID-19 or influenza.

More studies have reported higher COVID-19 related mortality among males compared to females. Corresponding to, that in the first half year of 2020, when COVID-19 evolved in Europe and all-cause mortality was substantially increased (Fig. 1), males had a markedly higher excess mortality compared to females (Table 2), according to data from 26 European countries providing data on weekly deaths counts to Eurostat, usable for the COVID-19 season.

Higher excess mortality among males compared to females was also observed in earlier seasons (Fig. 2). The season 2017/18 saw high excess mortality attributable to influenza^23^, and the cumulated sex-difference in excess mortality were of the same magnitude as during the COVID-19 pandemic (Table 2). Further, comparing the timing of peaks of increased excess mortality (Fig. 1) with peaks of low female excess mortality relative to male (Fig. 2), they seem to happen contemporarily. Indeed, there seems to be a general linear association between increasing overall excess mortality and sex-differential excess mortality (Fig. 3), being most pronounced among the 15 to 64 years old. This suggest that there is a consistent pattern of higher excess mortality among males in periods with overall excess mortality.

Many infectious diseases have been associated with sex-differences in cause-specific excess mortality e.g. bacteremia^24,25^, respiratory diseases including chronic obstructive pulmonary disease (COPD)^26^ and other viral infections^16,23,27^. On this basis, sex-differences in excess mortality during the COVID-19 pandemic should be understood in a broader context. Indeed, our finding suggest that higher male related excess mortality compared to females may be independent of what is causing the over-all increased mortality. The observed linear association between sex-differential and overall excess mortality may indicate that the female/male excess mortality incidence rate ratio will be constant and independent of size of overall excess mortality. Hence, a relative inequality in excess mortality between sexes is independent of size of the over-all excess, although the nominal difference between the sexes will increase with increasing over-all excess.

The general discrepancy in mortality between the sexes is well known. However, what is causing this discrepancy is still not understood and is probably due to many factors like behavioural and biological differences. Our finding of a u-shaped female/males excess mortality ratios with age corroborate the hypothesis that sex-hormone interactions with the immune system affect sex differences in mortality^11,12^. However, other conditions may also contribute, such as gender differences in health and hygiene behaviour^15^, including tobacco smoking^28^.

Our study is subject to limitations. Two countries, France and Montenegro, did not have sufficient data to be included in all seasons. However, all analyses were on mortality incidence rates, thus accounting for variations in participating populations over the seasons. Excluding the two countries from all analyses, did not affect the findings (data not shown). Furthermore, we used the EuroMOMO model to estimate the over-all baseline and the baselines for each of the sexes. The baseline estimation is based on a linear trend and a sine-curve seasonality over a 6 years period. The linear trend should compensate temporal changes in the background population. However, the assumption of a sine-curve seasonality may be a limitation. Although this is frequently applied in time-series analyses of mortality, it may represent a too rigid assessment of the seasonality.

The crude all ages sex-differences in excess mortality was not a priori expected to correspond to the sex-difference patterns observed in the age groups, due to different age distributions between the sexes. A reversion, as for example seen in the 2016/17 season, was not anticipated, which emphasize assessing differences in age distribution, when comparing populations.

In this study, we looked at excess mortality for each sex i.e. we exclude differences in the baseline or general mortalities, thereby excluding basic differences in mortality; which could eliminate the effect of some of the confounders in the over-all difference between the sexes. However, these confounders may also influence the sex-differential excess mortality. Our study is limited to European countries and the seasons 2016/7 to 2019/20, where the main causes to over-all excess mortality has been the infectious diseases influenza and COVID-19. Hence, our findings should not be extended to situation, where the cause to the over-all excess mortality not is an infectious disease. Further, excess mortality being an aggregated measure and as such it is neither predictive of individual outcomes, nor exempt from ecological fallacy.

We have analysed patterns in pooled European excess mortality. However, we found some variation between countries, including countries without a statistically significant difference between sexes, mainly countries with relatively low increases in excess mortality (data not shown).

## Conclusion

Under normal conditions female mortality will generally be lower than male mortality. In situations with excess mortality, e.g. due to winter circulation of respiratory pathogens, excess mortality increases more for males than for females. Because this was observed at similar magnitudes in seasons with influenza and with SARS-CoV-2, we suggest that this occurs independent of which pathogens are causing the excess mortality. Therefore, the sex-differences observed in COVID-19 associated deaths and in excess mortality during the COVID-19 pandemic does not represent a feature that is particular for the COVID-19 pandemic, but is associated to excess mortality in a more general way.

Sex-differentials in excess mortalities should be investigated further, especially as they may provide knowledge and tools supporting sex equality in public health management and prevention in situations with excess mortality.

## Supplementary Information

Supplementary Information 1.

Supplementary Information 2.
