# Supplementary material for: Sex-differences in COVID-19 associated excess mortality is not exceptional for the COVID-19 pandemic
Source: Sci Rep. 2021 Oct 21;11:20815. doi: 10.1038/s41598-021-00213-w (PMC8531278; doi:10.1038/s41598-021-00213-w)
Supplement: Supplementary file 1 — Supplementary Information 1. [file 41598_2021_213_MOESM1_ESM.docx]

**Supplementary 1**

Countries and seasons on which they had sufficient historical data on weekly numbers of all-cause deaths. Downloaded from Eurostat November 4, 2020.

| Country | Season (week 27 to week 26 the following year) | | | | |
| --- | --- | --- | --- | --- | --- |
|  | 2015/16 | 2016/17 | 2017/18 | 2018/19 | 2019/20 |
|  | Number of deaths* (thousands): Female / Male | | | | |
| Austria | 41.28 / 38.42 | 43.11 / 39.06 | 42.26 / 39.00 | 41.91 / 39.46 | 42.13 / 40.14 |
| Belgium | 54.80 / 53.01 | 56.34 / 54.02 | 55.91 / 53.94 | 55.46 / 53.18 | 59.41 / 56.48 |
| Bulgaria | 52.01 / 56.34 | 53.49 / 57.62 | 50.93 / 55.04 | 53.09 / 57.14 | 50.09 / 54.41 |
| Croatia | NA | 27.51 / 25.93 | 26.81 / 25.54 | 26.73 / 25.88 | 25.61 / 24.66 |
| Czech Republic | 53.62 / 55.43 | 54.92 / 56.32 | 55.02 / 56.48 | 54.56 / 56.83 | 54.51 / 57.07 |
| Denmark | 26.43 / 26.66 | 26.42 / 26.76 | 27.00 / 27.48 | 26.57 / 27.21 | 26.06 / 27.46 |
| Estonia | 7.97 / 7.26 | 8.28 / 7.42 | 8.28 / 7.28 | 8.08 / 7.27 | 8.16 / 7.19 |
| Finland | NA | 27.16 / 27.02 | 26.86 / 26.90 | 26.78 / 26.83 | 27.01 / 27.25 |
| France | NA | NA | NA | 303.41 / 301.13 | 313.77 / 311.26 |
| Hungary | NA | 67.83 / 64.56 | 66.08 / 62.89 | 67.64 / 64.09 | 64.48 / 61.10 |
| Iceland | NA | 1.15 / 1.17 | 1.10 / 1.17 | 1.09 / 1.10 | 1.14 / 1.15 |
| Latvia | NA | 15.13 / 13.42 | 15.17 / 13.60 | 14.82 / 13.24 | 14.50 / 12.56 |
| Liechtenstein | NA | 0.12 / 0.15 | 0.14 / 0.13 | 0.12 / 0.14 | 0.13 / 0.12 |
| Lithuania | NA | 20.70 / 20.03 | 20.73 / 19.06 | 20.24 / 18.58 | 19.44 / 18.60 |
| Luxembourg | NA | 2.05 / 2.09 | 2.12 / 2.19 | 2.08 / 2.15 | 2.09 / 2.21 |
| Malta | NA | 1.77 / 1.80 | 1.80 / 1.83 | 1.88 / 1.85 | 1.72 / 1.87 |
| Montenegro | NA | 3.14 / 3.54 | 3.06 / 3.29 | 3.15 / 3.44 | NA |
| Netherlands | NA | 77.98 / 72.66 | 78.64 / 74.10 | 76.25 / 73.24 | 80.77 / 79.38 |
| Norway | NA | 21.28 / 19.78 | 21.16 / 19.78 | 20.47 / 19.56 | 20.45 / 19.94 |
| Poland | NA | 194.13 / 206.44 | 196.58 / 210.69 | 197.49 / 210.91 | 195.01 / 209.45 |
| Portugal | NA | 55.95 / 55.59 | 55.87 / 56.85 | 55.85 / 55.51 | 56.16 / 56.43 |
| Serbia | NA | 52.67 / 52.54 | 49.80 / 49.96 | 50.76 / 51.42 | 49.07 / 50.21 |
| Slovakia | NA | 26.80 / 27.49 | 26.15 / 27.44 | 26.35 / 27.74 | 25.27 / 26.94 |
| Slovenia | NA | 10.22 / 10.12 | 10.23 / 9.88 | 10.48 / 10.19 | 10.36 / 10.04 |
| Spain | NA | 207.80 / 210.53 | 211.74 / 215.43 | 204.69 / 210.84 | 226.22 / 231.49 |
| Sweden | NA | 46.72 / 43.56 | 46.22 / 43.47 | 44.02 / 42.24 | 46.55 / 45.70 |
| Switzerland | 33.78 / 31.66 | 34.73 / 32.03 | 34.26 / 32.59 | 34.70 / 32.30 | 34.80 / 33.10 |

* NA indicate that data from the country in that season were missing or not sufficient i.e. data from five preceding years and the full actual season were not available.
