# Supplementary material for: Sex-differences in COVID-19 associated excess mortality is not exceptional for the COVID-19 pandemic
Source: Sci Rep. 2021 Oct 21;11:20815. doi: 10.1038/s41598-021-00213-w (PMC8531278; doi:10.1038/s41598-021-00213-w)
Supplement: Supplementary file 2 — Supplementary Information 2. [file 41598_2021_213_MOESM2_ESM.docx]

**Supplementary 2**

Linear association between differences in excess mortality (females – males) and overall excess mortality:

mr_f_ – mr_m_ = α * mr_overall_ (1)

where mr_f_ , mr_m_ and mr_overall_ are mortality incidence rates for females, males and overall, respectively.

With d_f_ and d_m_ as number of female and male deaths, and y_f_ and y_m_ the corresponding time or persons at risk, the mortality rates will be: mr_f_ = d_f_ / y_f_ , mr_m_ = d_m_ / y_m_ and mr_overall_ = (d_f_ + d_m_) / (y_f_ + y_m_). Which, inserted in the linear association above, gives:

d_f_ / y_f_ – d_m_ / y_m_ = α * (d_f_ + d_m_) / (y_f_ + y_m_)

((y_f_ + y_m_) / y_f_) * d_f_ – ((y_f_ + y_m_) / y_m_) * d_m_ = α * d_f_ + α * d_m_

(((y_f_ + y_m_) / y_f_) – α) * d_f_ = (((y_f_ + y_m_) / y_m_) + α) * d_m_

d_f_ / d_m_ = (((y_f_ + y_m_) / y_m_) + α) / (((y_f_ + y_m_) / y_f_) – α)

d_f_ / d_m_ = (y_f_ / y_m_ + 1 + α) / (y_m_ / y_f_ + 1 – α) (2)

or as female/male mortality rate ratio FMRR:

(d_f_ / y_f_) / (d_m_ / y_m_) = (y_m_ / y_f_) * (y_f_ / y_m_ + 1 + α) / (y_m_ / y_f_ + 1 – α) (3)

mr_f_ / mr_m_ = (y_f_ + (1 + α) * y_m_) / (y_m_ + (1 – α) * y_f_)

FMRR = (y_f_ + (1 + α) * y_m_) / (y_m_ + (1 – α) * y_f_) (4)

The equations 2 and 3 reveals that the exact times or persons at risk for females and males are not needed, the ratio between them is sufficient.

From 4, it easily follows that

α = ((FMRR – 1) * y_f_ + (FMRR – 1) * y_m_) / (y_m_ + FMRR * y_f_)

Hence:

mr_f_ = mr_m_ <=> α = 0 <=> FMRR = 1

mr_f_ < mr_m_ <=> α < 0 <=> FMRR < 1

mr_f_ > mr_m_ <=> α > 0 <=> FMRR > 1
